# Editorial: Neurocognitive disorders and depression—Complex interrelationships

**DOI:** 10.3389/fpsyg.2023.1088086

**Published:** 2023-01-30

**Authors:** Katarzyna Milana Broczek, Marie-Christine Gely-Nargeot, Pietro Gareri

**Affiliations:** ^1^Polish Society of Gerontology, Mazovia Branch, Warsaw, Poland; ^2^Université Paul Valéry, Montpellier III, Montpellier, France; ^3^Dipartimento delle Cure Primarie, ASP Catanzaro, Catanzaro, Italy

**Keywords:** cognitive impairment, dementia, depressive symptoms, biomarkers, neuroimaging, caregivers, COVID-19

Cognitive impairment and depressive symptoms often coexist especially in older adults, however depression may be associated with signs of cognitive decline at any age. The complex relationships between cognitive disorders and depression may be viewed from biological, neuropsychological, medical, and social perspectives. Untangling these interrelationships may facilitate diagnostic precision, choice of individually tailored treatment options, and development of practice oriented recommendations.

There are many trajectories linking depression and cognitive decline. Depressive symptoms might precipitate the onset of Alzheimer's disease (AD) and other dementias, depression may also develop in the course of previously diagnosed neurocognitive disorder due to neurodegenerative or vascular causes. Cognitive decline resulting from major depression is often related to as “pseudodementia” and cognitive problems diminish over the course of antidepressant treatment. On the other hand, depressive disorders increase the risk for cognitive impairment in the future.

In older adults, mental health problems are often underdiagnosed and undertreated, therefore societies such as Alzheimer's Association^1^ and Alzheimer's Europe^2^ promote early diagnosis of depressive symptoms in individuals with Alzheimer's disease.

The optimal therapeutic approach to neurocognitive disorders and depression includes medications, diet, psychological therapy, art therapy, and social support. Increasing evidence points to treatments based on understanding underlying mechanisms (e.g., increased inflammatory status) such as probiotics (Dobielska et al., 2022).

The Research Topic addresses the issue of neurocognitive disorders and depression from various and exciting standpoints. It includes six original research articles, one review, and two systematic reviews written by 61 outstanding authors.

Masse et al. present a very interesting and useful approach to differentiating results of neuropsychological assessment between normal aging, late life depression (LLD) and mild AD including the following domains: verbal episodic memory, executive skills, mental processing speed, constructional praxis, and semantic memory. Impairment in one cognitive domain was relatively frequent in healthy older adults, low scores in two domains prevailed in LLD, while decline in at least three domains was characteristic for AD. These findings provide important clues for clinical practice such as regular follow-up of cognitive performance in patients with LLD.

Van den Bossche and Schoenmakers analyzed affiliate stigma in relatives of people living with dementia diagnosis and its impact on caregivers' mental health. Interestingly, many caregivers' characteristics including age, sex, and education were correlates of the impact of stigma on caregivers' life. The results of the study are important for creating an inclusive environment for people living with dementia and their family members.

Cao et al. report on the psychological consequences of acute myocardial infarction treated with percutaneous coronary intervention. Of note, symptoms of post-traumatic stress disorder were present in substantial percentage of patients 3 months after the event.

Hall et al. studied cognitively normal older adults in terms of neurobehavioral symptoms known as risk factors for cognitive decline such as depression, apathy, anxiety, worry, and disordered sleep in relationship to blood-based biomarker of neurodegeneration (plasma total tau, t-tau). T-tau was not a predictor of any of the assessed symptoms, however, additional analysis revealed that in individuals with the highest quintile of t-tau, the above neurobehavioral symptoms were significantly related to the biomarker level. These results are important clues for further research, as blood-based biomarkers are an exciting alternative to cerebro-spinal fluid (CSF) and PET imaging assessment of tau burden in the brain.

Egglefield et al. applied magnetic resonance imaging to assess cortical thickness and hippocampal volume in patients with or without vascular changes in the brain and undergoing pharmacological treatment for depression. Vascular depression (VD) was defined as presence of deep white matter hyperintensities (DWMHs) on T2-weighed FLAIR sequence. Interestingly, no differences were found between VD and non-VD in terms of gray matter characteristics.

Wang et al. assessed psychological mechanisms underlying depression in young adults with special consideration of resilience, attentional bias, and neuroticism. Depressive symptoms were frequent in college students which indicated a urgent need for preventive and therapeutic strategies to promote mental health across lifespan.

The review by Hammar et al. focuses on neurocognitive profiles of major depressive disorder. The authors describe three hypotheses crucial to understanding cognitive decline in depression, namely state, scar, and trait hypotheses. Clinical implications of residual cognitive symptoms as well as potential preventive strategies are discussed.

Ma et al. provide thorough insight into relationship between interleukin 6 (IL-6) level, depression, and cognitive-behavioral therapy (CBT) for depression. The authors analyzed the results of 10 studies and found that peripheral IL-6 levels were significantly lower after CBT. The authors discuss potential modulating anti-inflammatory effects of CBT.

Carbone et al. provide a systematic review on psychological consequences of COVID-19 pandemic for people living with dementia and their caregivers. As suspected most studies under review showed increased psychological burden and physical strain in caregivers as well as increased anxiety and general decline, fatigue, and cognitive impairment in people living with dementia. Severely decreased access to health care and social services due to lockdown was definitely among culprits of psychological consequences of the pandemic. A positive notion is related to technological advances enabling video-consultations.

COVID-19 pandemic poses significant threats to health care systems, but also opens new possibilities of integrated care. How these options will develop in the future and whether the post-COVID era will be friendly toward people suffering from neurocognitive disorders and depression is an open question.

## Author contributions

KB is the lead author of the manuscript, PG and M-CG-N commented and revised the draft. All authors approved the final version of the manuscript.

## Footnotes

^1^ https://www.alz.org

^2^ https://www.alzheimer-europe.org

## References

[B1] Dobielska M. Bartosik N. K. Zyzik K. A. Kowalczyk E. Karbownik M. S. (2022). Mechanisms of cognitive impairment in depression. May probiotics help? Front. Psychiatry 13, 904426. 10.3389/fpsyt.2022.904426 35757204 PMC9218185

